# The Role of Nutrition in the Development and Management of Chronic Obstructive Pulmonary Disease

**DOI:** 10.3390/nu16081136

**Published:** 2024-04-11

**Authors:** Allison Heefner, Tijana Simovic, Kasey Mize, Paula Rodriguez-Miguelez

**Affiliations:** 1 Department of Kinesiology and Health Sciences, Virginia Commonwealth University, Richmond, VA 23284, USA; 2 School of Medicine, Virginia Commonwealth University, Richmond, VA 23284, USA; 3 Division of Pulmonary and Critical Care, Virginia Commonwealth University, Richmond, VA 23284, USA

**Keywords:** COPD, diet, nutrition, fiber, vitamins, polyphenols, alcohol, iron, nitrates

## Abstract

Chronic obstructive pulmonary disease (COPD) is a prevalent lung condition associated with significant morbidity and mortality. The management of COPD classically involves pulmonary rehabilitation, bronchodilators, and corticosteroids. An aspect of COPD management that is currently lacking in the literature is nutritional management, despite the prevalence of inadequate nutritional status in patients with COPD. In addition, certain nutritional imbalances have been reported to increase the risk of COPD development. This review summarizes the current literature on the role diet and nutrients may play in the risk and management of COPD development.

## 1. Introduction

Chronic obstructive pulmonary disease (COPD) is a common lung condition with a prevalence of more than 212 million cases and 3.3 million deaths worldwide [1]. COPD is caused by irreversible airflow obstruction [2,3,4] due to chronic bronchitis and/or emphysema [2]. Common symptoms include dyspnea [2,5], cough [2,5], increased sputum production [2,5], and decreased exercise tolerance [2,3,4,5,6]. The most commonly cited risk factor for the development of COPD is cigarette smoke [2,7,8]. However, never-smokers make up a substantial portion of patients with COPD [9]. Indeed, it is estimated that up to half of COPD cases worldwide are due to non-tobacco causes [10]. This has led to investigations into other factors that may contribute to and/or accelerate COPD development, including dietary imbalances [5,8,11,12].

Weight loss and appetite suppression occur in nearly half of patients with COPD [13], making malnutrition a common comorbidity [14]. Multiple factors precipitate this, including heightened physical demands of ventilation and increased sedentary behaviors [15]. Consequently, individuals with COPD scarcely obtain adequate amounts of micronutrients [16]. Despite this, nutritional research in patients with COPD is lacking.

In this narrative review, we will summarize the data on various nutrients, diets, and supplements in the prevention and management of COPD (Table 1). This review was conducted via a literature search on PubMed using the keywords “nutrition”, “nutrients”, “diet” “COPD”, “emphysema”, “bronchitis”, and “health outcomes”. Due to the paucity of literature regarding nutrition and COPD, studies from the late 1990s to present day were included.

## 2. Meat

Most studies investigating the link between COPD development and meat consumption focus on processed red meat (i.e., bacon, ham, sausage, and luncheon meat). Growing evidence suggests an association between processed meat consumption and the risk of COPD [17,18,19,20], with a high intake (greater than or equal to 75 g/week) of processed red meat associated with a 40% higher risk of COPD [21]. Indeed, each additional 50 g per week of processed red meat intake is associated with an 8% higher risk of COPD development [21]. Moreover, processed red meat, but not unprocessed (i.e., pork, beef/veal, and minced meat), may be associated with a higher incidence of COPD development at 13-year follow-up [22].

### 2.1. Health Outcomes

Studies investigating the relationship between meat consumption and health outcomes in patients with COPD are scarce. Frequent processed meat intake was associated with decreased lung function [force expiratory volume in one second (FEV_1_) [20,23], forced vital capacity (FVC) [23], and forced expiratory volume in one second over forced vital capacity (FEV_1_/FVC) [20,23] in the general population. Meanwhile, in patients with COPD (post-bronchodilator FEV_1_/FVC lest than or equal to 0.70), one study identified high processed meat consumption as a risk factor for COPD-related hospital readmissions [24]. As far as we know, no studies have investigated the associations between meat intake and lung function or the potential health benefits of decreasing processed meat intake in patients with COPD.

### 2.2. Mechanisms

The primary mechanism explaining the relationship between processed meat intake and COPD development is currently unknown; however, some potential mechanisms have been proposed. Processed meats contain high amounts of nitrates and nitrites [25], which are added to meat as preservatives and color additives [26]. Nitrates from processed meat are commonly converted to nitrosamine compounds that are involved in the formation of reactive nitrogen species and may amplify oxidative stress and inflammatory processes [27], two keystones of COPD pathobiology [28]. Indeed, preclinical studies associated long-term dietary nitrate intake with the development of pulmonary emphysema [29,30]. Additionally, processed meats contain high amounts of saturated fatty acids and advanced glycation end products [31], which are known to stimulate systemic inflammation [32] and increase oxidative stress [32]. Of note, a similar pro-inflammatory profile [33] was identified in patients with COPD following the consumption of processed red meat [34]. Lastly, diets rich in meat products have also been linked to increased levels of trimethylamine N-oxide (TMAO) [35,36]; elevated circulating levels of this byproduct have been associated with all-cause mortality in COPD [37]. The above mechanisms highlight the negative role of inflammation on COPD health outcomes. Indeed, COPD is characterized by an amplification of the normal inflammatory response, resulting in multisystemic changes in the lungs [38]. Furthermore, the heightened inflammatory response in COPD is thought to help drive the common comorbidities—such as heart disease, muscle wasting, and diabetes—seen in this population [38].

### 2.3. Recommendations

Existing evidence has shown the negative health effects of processed meat in the general population [39]. In COPD, an increased disease development risk [21] as well as worse health outcomes [24] have been related to increased meat consumption, supporting the concept that patients with COPD may benefit from limiting processed meat consumption. Additionally, those at risk of COPD may specifically benefit from reducing their meat intake to no greater than 75 g/week. However, information is still limited, and more studies are needed to establish specific guidelines.

## 3. Fruit and Vegetables

Due to their antioxidant capacity, diets rich in fruits and vegetables have been proposed to be protective against COPD [40]. Indeed, people who consume diets rich in fruit and vegetables are at a lower risk of COPD development [40,41,42,43,44]. However, some conflicting results have been reported, suggesting that only the long-term intake of fruits, but not vegetables, may be linked to a lower risk of COPD [45].

### 3.1. Health Outcomes

In the general population, a diet rich in antioxidants and/or a diet rich in fruit and vegetables is associated with better lung function, specifically FEV_1_ [23,44]. One study in individuals with chronic airflow limitation (COPD, asthma, and COPD plus asthma) identified a positive association between serum antioxidants and pulmonary function (FEV_1_ percent predicted and FVC percent predicted) [46]. Additionally, a three-year prospective study demonstrated that increasing the consumption of fresh fruits and vegetables improved lung function (specifically FEV_1_) in patients with COPD (GOLD stage I–IV) [47].

### 3.2. Mechanisms

Fruits and vegetables are rich in vitamin C [48], vitamin E [49], and β-carotene [50], all of which are described to have antioxidant and anti-inflammatory properties [51,52] and, thus, protect against oxidative stress, one of the root causes of COPD. Indeed, dietary antioxidant intake has been shown to lower oxidative stress [53]. However, increasing the intake of fruits and vegetables had no effect on biomarkers of airway systemic inflammation or oxidative stress in patients with moderate-to-severe COPD [54].

### 3.3. Recommendations

Current evidence from the limited number of observational studies infers that increased fruit and vegetable intake may be beneficial for people with COPD, improving pulmonary health outcomes, which is possibly related to their antioxidant and anti-inflammatory capacity. However, clinical trials in this area are minimal, and future studies should investigate the potential benefits that greater fruit and vegetable consumption have in this population. Thus, a recommended increase in daily fruits and vegetables may be beneficial for patients with COPD.

## 4. Dietary Fiber

Dietary fiber consumption is associated with positive effects on the metabolic [55] and cardiovascular [56] systems. In terms of COPD, high total dietary fiber intake was associated with the reduced risk of COPD development [57,58,59,60]. Specifically, long-term high dietary fiber intake (greater than or equal to 26.5 g/day) was associated with a 30% lower risk of COPD, while increasing total dietary intake by 1 g/day was associated with a 5% risk reduction [60]. When investigating sources of dietary fiber, total cereal and fruit, but not vegetable fiber, have been related to a lower risk of COPD [61].

### 4.1. Health Outcomes

To the best of the authors’ knowledge, no specific information is available evaluating dietary fiber intake and health outcomes in COPD. However, in the general population, better lung function (FEV_1_, FVC, FEV_1_/FVC) has been related to higher fiber intake [59,62]. Interestingly, decreasing total fiber intake has been associated with obstructive airflow patterns [63].

### 4.2. Mechanisms

Although the mechanism connecting dietary fiber and risk of COPD is unknown, the positive benefits from greater dietary fiber intake in the general population have been postulated to be related to improved gut microbiome and reduced inflammatory responses [64]. Fiber fermentation results in the formation of metabolites such as short-chain fatty acids [65], which are known to have systemic [66] and pulmonary anti-inflammatory effects [67]. Diets low in fiber can also result in gut dysbiosis and promote chronic, systemic inflammation [68].

### 4.3. Recommendations

Despite the established benefits of dietary fiber intake on cardiovascular [69] and metabolic [55] health, its potential role in lung health is largely underexplored. High dietary fiber consumption may exhibit some benefits for people with COPD, although further research exploring fiber consumption and health outcomes in this population is needed. Therefore, current recommendations support the concept that those at risk of COPD should consider incorporating greater than or equal to 26.5 g/day of fiber into their diet. Given the lack of research on dietary fiber and COPD health outcomes, no recommendation regarding high fiber diets for patients with COPD can be made at this time.

## 5. Vitamin D

In accordance with the American Association of Clinical Endocrinologists, vitamin D deficiency is defined as a serum level of 25-hydroxyvitamin D (25(OH)D) lower than 75 nanomolar per liter (nmol/L) [70]. By this definition, as much as 23% of the United States population is at risk of vitamin D deficiency [71]; vitamin D deficiency is frequently identified in patients with COPD [72,73,74,75,76]. The etiology of this deficiency in COPD seems to be multifactorial, including poor dietary intake [77], reduced exposure to sunlight [77], catabolism by common medications prescribed for the management of COPD (i.e., glucocorticoids) [77], or the effects of comorbid conditions such as chronic kidney disease [77].

### 5.1. Health Outcomes

Vitamin D deficiency has been identified as a risk factor for the development of COPD [72,78,79], specifically levels lower than 32 nmol/L, which have been associated with a 23% increased risk of developing COPD [72]. Multiple studies have found that vitamin D deficiency is linked with poor lung function in patients with COPD (GOLD stage I–IV) as measured by FEV_1_ [76,80] and FVC [76,77]. Additionally, vitamin D supplementation decreased the rate of acute pulmonary exacerbations [74,81,82] in patients with mild-to-very severe disease. Despite these findings, some questions have arisen as to whether vitamin D supplementation improves lung health outcomes primarily due to issues replicating these findings [8,73]. A recent meta-analysis compiling 19 studies and more than 2000 patients supported the relationship between vitamin D supplementation and lung function improvements, specifically in COPD [81]. To note, there are data to support the concept that vitamin D deficiency may play a role solely in the development of emphysema and no other COPD phenotypes. Therefore, it is plausible that studies that did not find benefit in vitamin D supplementation consisted of patients with COPD whose disease state was more attributable to chronic bronchitis rather than emphysema [83]. Besides lung health, vitamin D deficiency has also been linked to an increased risk of mortality in people with COPD [72]. Indeed, vitamin D levels lower than 32 nmol/L were associated with a 38% increased risk of overall mortality and 57% greater COPD-specific mortality when compared to patients with COPD with higher vitamin D levels [72].

### 5.2. Mechanisms

The exact mechanisms linking vitamin D deficiency and COPD are largely unknown [77]. Because of the high prevalence of vitamin D deficiency [71] and its effects on gene regulation and immune cell defense [83], researchers have explored the effects of vitamin D on respiratory diseases such as asthma [84] and COPD [81]. As previously mentioned, patients with COPD have systemic inflammation [2,8,85], and serum 25(OH)D concentrations are negatively associated with inflammatory biomarkers in COPD [85]. Cigarette smoke also inhibits vitamin D receptor translocation which leads to the downregulation of vitamin D signaling [86] and may contribute to promoting a proinflammatory environment in the airways [81]. Indeed, an animal model of vitamin D receptor deficiency exhibited increased inflammation in the lungs [87]. Similarly, another preclinical study showed that vitamin D inhibited alveolar macrophage proliferation and the associated inflammatory response [83]. Therefore, it is postulated that vitamin D deficiency would disrupt this balance of inflammatory control in the lungs, leading to tissue destruction [83].

### 5.3. Recommendations

The American Association of Clinical Endocrinologists defines optimal serum vitamin D levels as those greater than 75 nmol/L [70], although that cutoff is not agreed upon by the literature nor regulatory agencies [70,88]. However, evidence supports that serum levels of 25(OH)D of 50 nmol/L or greater may reduce mortality from respiratory diseases [89], and levels above 55 nmol/L were linked to the greatest reduction in risk of COPD development [72]. Indeed, multiple studies recommend considering vitamin D supplementation in patients with COPD [11,73,75], with some studies finding optimal serum 25(OH)D levels as those greater than 50 nmol/L [72,73,89]. Furthermore, the Global Initiative for Chronic Obstructive Lung Diseases recommends that all patients with COPD hospitalized for exacerbations are evaluated for severe vitamin D deficiency and treated with appropriate supplementation [2]. The specific dosing of vitamin D should be determined based on the individual patient’s serum 25(OH)D levels and their risk factors that may affect vitamin D production, bioavailability, and/or catabolism [88]. Although vitamin D toxicity is rare [90], it is not recommended to exceed serum 25(OH)D levels greater than 374 nmol/L [88]. Given the decreased risk of COPD development and the potential decreased risk of COPD disease progression, individuals at risk for COPD and those with COPD may consider vitamin D supplementation with a goal serum 25(OH)D level equal or greater than 55 nmol/L.

## 6. Vitamins A, B, C, and E

Multiple types of vitamins have been shown to have beneficial pulmonary effects in the general population [8]. However, it has recently been identified that people with COPD consume fewer vitamins than recommended [91,92] and present with lower circulatory concentrations of vitamins A, C, and E when compared to the general population [93].

### 6.1. Health Outcomes

There is minimal research available on the health benefits of vitamin supplementation in patients with COPD. However, dietary intakes of vitamin A [94,95], vitamin C [96], and vitamin E [97,98] have been associated with better lung function in the general population [99].

The effect of vitamin A supplementation on COPD symptomatology and disease progression is not well understood. Early evidence has suggested that vitamin A deficiency may contribute to, or even increase, the severity of respiration dysfunction in COPD [100], with low serum concentrations of vitamin A identified in patients with COPD experiencing an exacerbation [101]. Additionally, a randomized control trial of oral vitamin A supplementation for 30 days in patients with mild-to-moderate COPD identified improvements in lung function (FEV_1_) [95], supporting the relationship between vitamin A and pulmonary health.

Regarding vitamin B, it has been suggested that deficiencies in vitamin B6 may be associated with a higher risk of frailty in patients with COPD [102], although no associations have been identified with other vitamins from the B family, including B1, B2, B3, B9, or B12 [102]. In this line, evidence also supports that a combination of pulmonary rehabilitation with daily oral B12 supplementation in people with moderate-to-severe COPD led to minor improvements in exercise time [103].

Other vitamins such as C and E have also been proposed to exert a beneficial effect in people with COPD. For example, an increase in dietary vitamin E has been positively associated with a lower risk of COPD [104] and better lung health (FEV_1_ and FVC) [104,105]. However, conflicting evidence also exists [105,106], with some studies identifying no improvements in lung health when compared to traditional treatments [107]. In regard to vitamin C, multiple studies have shown a positive association between vitamin C intake and pulmonary health, specifically FEV_1_ [108,109,110,111]. Similarly, people that use combustible tobacco and consume a diet rich with vitamin C exhibit slower rates of pulmonary decline (FEV_1_) when compared to those with low vitamin C intake [112]. This finding was confirmed in patients with COPD, with daily doses of 400 milligrams of vitamin C associated with better lung function (FEV_1_ percent predictive and FEV_1_/FVC) [15], and daily doses of 2 g/day were associated with fewer pulmonary exacerbations over a six-month period [113]. Additionally, significant improvements in antioxidant capacity and vascular function were also observed in patients with COPD (GOLD stage I–IV) after an acute dose of a combination of vitamin C, vitamin E, and α-lipoic acid [114].

### 6.2. Mechanisms

Considering the connection between air pollution, tobacco smoke, oxidative stress, and COPD, it is not surprising that vitamins, due to their antioxidant properties helping to mitigate free radicals and minimize oxidative stress [115,116], would exhibit beneficial effects in this population. For example, supplementation with vitamin B6 has been linked to reduced oxidative damage and, therefore, has been suggested as beneficial for the treatment of chronic pulmonary diseases [117]. Similar properties have been associated with vitamin A, which may play a role protecting lung epithelial cells from irritants [118]. Additionally, vitamin C may also have positive effects on vascular health [114,119,120], which is frequently impaired in patients with COPD. Thus, vitamin supplementation may be beneficial in patients with COPD by restoring antioxidant balance, reducing oxidative stress, and preventing further tissue damage [121].

### 6.3. Recommendations

At this time, not enough research has been conducted on vitamin A, B, and E supplementation to define dosage recommendations. The role of these vitamins in COPD should be further investigated due to their potential benefits such as reducing oxidative stress [122] and protecting the airways [118,119,123]. In regard to vitamin C, a meta-analysis of ten randomized controlled trials including nearly 500 participants showed a significant improvement in lung function in individuals that supplemented 400 milligrams of oral vitamin C per day [15]. Additional benefits with fewer pulmonary exacerbations have been described in patients with COPD that received two grams of daily oral vitamin C supplementation [113]. In general, vitamin C poses few health risks and has been deemed safe to consume with no lasting health consequences, even in circumstances where extremely high concentrations are ingested in a single dose [124]. However, regular oral intake of greater than one gram of vitamin C per day is not recommended due to an increased risk of renal calculi formation [124]. Therefore, based on the current literature, oral vitamin C supplementation may be incorporated into COPD management at a dose between 400 and 1000 milligrams per day to balance the benefits of supplementation with the risks of toxicity. Recommendations regarding vitamins A, B, and E cannot be made at this time given the paucity of available data.

## 7. Iron

Non-anemic iron deficiency is common in people with COPD [125,126,127,128] and has been linked to reduced exercise tolerance [129]. It is well known that exercise tolerance is diminished in patients with COPD [2,3,6,125,128], negatively impacting their quality of life [6,125]. Indeed, iron supplementation should be of interest in COPD, as it has been shown to increase exercise tolerance in other chronic conditions such as heart failure [130]. However, there remains a paucity of clinical trials establishing the utility of iron replacement in patients with COPD [127].

### 7.1. Health Outcomes

Iron deficiency has been established as common in patients with obstructive pulmonary disease [125,126,127,128] and has even been identified as a risk factor for the development of COPD [131]. Once pathology is established, iron deficiency without anemia in moderate-to-severe COPD is connected to more hypoxemia/dyspnea [126,129], higher levels of inflammatory markers such as C-reactive protein [126], and decreased exercise tolerance [129] when compared to patients with COPD who are iron replete [126,129]. To note, few studies have investigated the effects of iron repletion on patients with COPD.

### 7.2. Mechanisms

The exact mechanism linking iron supplementation and improved outcomes in COPD is unknown. The prevailing theory involves a reduction in systemic inflammation [125,126,128], diminishing iron deficiency, and skeletal muscle dysfunction [127,129,132], which are all contributors to reduced exercise tolerance [127,128,132]. Of note, systemic inflammatory diseases elevate hepcidin levels, a peptide hormone involved in the regulation of iron [125,133], which cause a decrease in serum iron [126] due to decreased dietary iron absorption [133] and by sealing iron away in macrophages during inflammatory states [133]. Indeed, hepcidin levels were significantly higher in patients with iron deficiency and COPD compared to people with iron deficiency without COPD [126], illustrating the role of systemic inflammation in increasing hepcidin levels [126]. Decreased serum iron and dietary iron absorption [126,133] can also lead to iron deficiency, affecting exercise tolerance, as iron is critical to skeletal muscle function [127,128,129,132]. In this line, one study showed better exercise tolerance after intravenous iron supplementation in patients with COPD as independent of hemoglobin [128].

### 7.3. Recommendations

Given the scarcity of trials investigating the effects of iron supplementation on COPD, recommendations cannot be made at this time [128]. However, a growing body of literature is encouraging the need for larger-scale trials to investigate the effect of iron supplementation on exercise tolerance in COPD [127,128,132]. To the best of our knowledge, all studies to date used intravenous iron [125,127,128,132] which was well tolerated [125,127,128,132], although hypophosphatemia [132], a well-known side effect of intravenous iron therapy [132], was reported in one of the trials. Overall, iron supplementation in non-anemic iron-deficient patients with COPD may have the ability to improve exercise tolerance with a good safety profile. Although promising, no recommendations at this time can be suggested regarding intravenous iron therapy as part of COPD’s standard of care given a paucity of data, documented side effects, the burden of infusions, and the risk of repeated intravenous access needed for infusions.

## 8. Nitrates

Dietary nitrate supplementation has been shown to have beneficial effects, primarily related to physical performance in both healthy [134] and disease states [3,4,6,135], including peripheral artery disease [136], hypertension [137], and heart failure [137,138,139], although limited information is available with regard to COPD. Nitrate supplementation, primarily through beetroot juice, has been tied to improvements in vascular function [140] and exercise tolerance [136], two cardinal features of COPD.

### 8.1. Health Outcomes

Beetroot juice supplementation has been shown to increase exercise tolerance [3,4,6,135] and vascular endothelial function [6,135] in patients with COPD (GOLD stage I–IV and II–IV, respectively). Although the value of beetroot juice supplementation has been debated in the literature due to contradictory findings [12,141,142], a recent trial showed the positive effects of beetroot juice supplementation on exercise tolerance in patients with COPD (GOLD stage II–IV) [6]. Of note, results regarding beetroot juice supplementation may be partially attributable to the length of supplementation. Studies finding beneficial effects of beetroot juice supplementation on exercise tolerance used short-term usage (acute [4,135] or at least two weeks of supplementation [3,6]), whereas studies not identifying beneficial effects of beetroot juice supplementation had subacute supplementation (schedules not defined by acute or chronic) [143,144,145]. This discrepancy emphasizes the need for further studies on the effect of the duration and timing of beetroot juice supplementation on vascular function and exercise tolerance in patients with COPD.

### 8.2. Mechanisms

The effects of beetroot juice supplementation are associated with its high organic nitrate (NO_3_^−^) content [146] that, once consumed, is reduced to nitrite (NO_2_^−^) in the oral cavity and then to nitric oxide (NO) in the stomach, where it is then absorbed [146]. NO is a potent vasodilator [141,146] that plays a role in the regulation of blood flow [3,4], mitochondrial biogenesis [146], mitochondrial respiration [146], glucose uptake [135,146], and muscle relaxation [141,146]. Although the exact mechanism linking the benefits of dietary nitrate supplementation and exercise tolerance is unclear [4], it is thought that the etiology is multifactorial and includes some or all the effects described above [4,146].

Endothelial dysfunction is defined by a reduced vasodilatory state [147], characterized by an imbalance of vasodilation and vasoconstriction [147,148]. An important component of vascular endothelial-dependent vasodilation is NO synthesis and bioavailability, and reductions in this vasodilator are associated with poor dilatory response [148]. Therefore, it is plausible that dietary nitrate supplementation may enhance vascular function via increasing the availability or synthesis of NO [135]. Indeed, both acute and chronic supplementation with beetroot juice increases plasma nitrate levels in patients with COPD [3,4,143].

### 8.3. Recommendations

Beetroot juice supplementation in patients with COPD has the potential to benefit both vascular dysfunction [6,135] and exercise tolerance [3,4,6,135]. The largest trial of beetroot juice supplementation in patients with COPD (GOLD stage II–IV) provided 12.9 millimolar of nitrate, twice weekly, for eight weeks, in combination with pulmonary rehabilitation with no adverse events identified [6], as confirmed by other trials [141]. Considering the low risk of this type of supplementation and the potential to increase exercise tolerance [3,4,6,135] and vascular function [6,135], beetroot juice may exert positive effects in patients with COPD, although further trials are warranted to identify the dosage, timing, and frequency that would allow patients to gain maximal benefit. Thus, weekly supplementation with beetroot juice at 12.9 millimolar of nitrate for eight weeks may offer benefits to patients with COPD. Recommendations about longer supplementation with beetroot juice cannot be made at this time due to a lack of long-term studies.

## 9. Other

### 9.1. Alcohol

Very little is known about the effects of alcohol intake on COPD. High alcohol intake has been identified in the COPD population, with higher odds of exceeding both daily and weekly alcohol recommendations [149]. Indeed, the heavy use of alcohol increases the risk of COPD development when compared to moderate use [150]. In addition, heavy alcohol intake has been associated with an accelerated decline in lung health (FEV_1_ and FVC) in the general population [151]. Similar findings have been observed in people with COPD with low overall alcohol consumption (defined as three or less drinks per day), correlating with better lung function (FEV_1_ and FEV_0.75_) [152]. Interestingly, several studies have shown that pulmonary function is higher in patients with COPD who occasionally or lightly consume alcohol when compared to non-drinkers [42,152], which is potentially associated with the antioxidant effects [150] and inhibition of proinflammatory molecules [152] by certain types of alcohol. However, high alcohol consumption has not been shown to have beneficial effects on COPD development nor disease course [42,150,152], and these results should be interpreted with caution. Thus, heavy alcohol use should be avoided in those with COPD, and no recommendations can be made at this time about low-to-moderate alcohol consumption.

### 9.2. Polyphenols

Polyphenols are plant-derived compounds with potential antioxidant and anti-inflammatory effects [153]. Considering that COPD is characterized by a pro-oxidative [2], pro-inflammatory state [154] and, consequently, a reduced antioxidant capacity [155], there has been interest in exploring the potential therapeutic benefit of polyphenols on the pathogenesis and disease course of COPD. For example, polyphenol intake has been shown to reduce the risk of developing COPD [156,157] and, once the disease is developed, may reduce lung inflammation [158] and markers of cardiovascular disease risk [159]. Indeed, six-month supplementation with quercetin—a plant flavonoid—significantly reduced proinflammatory biomarkers in the bronchoalveolar lavage of patients with COPD (GOLD stage II–III) [158], while six-week supplementation with resveratrol—a stilbenoid polyphenol [160]—resulted in improvements in arterial stiffness, myocardial perfusion, and distance walked in patients with COPD (GOLD stage II–IV) [159].

Preliminary evidence supports polyphenols’ role in reducing the risk of COPD development [156,157], as well as health benefits once the disease is established [158,159]. However, as the literature stands, there is not enough evidence to recommend the dosage or type of polyphenol intake for patients with COPD, nor for the prevention of this disease, due to the heterogeneity of these studies and minimal information [157,159,161]. Notwithstanding, a diet rich in polyphenols may be beneficial to those at risk of developing COPD due to their potential anti-inflammatory effects [158]. Although there is a paucity of safety data on specific types and dosages of polyphenols, a habitual diet is unlikely to have the dose of polyphenol intake necessary to cause adverse effects [162]. Currently, no recommendations can be made regarding a dietary increase in polyphenols nor polyphenol supplementation for patients with COPD due to a lack of studies and safety data.

### 9.3. Dietary Patterns

Some studies investigated the relationship between dietary patterns (i.e., Western- vs. Mediterranean-style diet) and the risk of COPD. It was identified that the prudent dietary pattern (diets rich in fruits, vegetables, and whole grains) was associated with a lower risk of COPD [44], while Western-style diets (diets rich in processed meats, refined grains, desserts, and sweets) were associated with a higher risk of COPD [163,164]. Additionally, the interplay between a diet high in processed meat intake and other lifestyle behaviors, such as smoking and an overall unhealthy diet, have been linked to yielding the highest hazard ratio for COPD development [19]. Adhering to the Dietary Approaches to Stop Hypertension (DASH) diet (rich in fruits, vegetables, and grains and low in sodium, fatty meats, and sugar) was inversely associated with the risk of COPD [165], while mixed results regarding a Mediterranean diet (high intakes of vegetables, legumes, fruits, nuts, grains, fish, seafood, and extra virgin olive oil and a moderate intake of red wine) have been reported [165,166]. Currently, there is not enough information available to recommend a specific dietary pattern for patients with COPD. However, guidelines for the general population encouraging adherence to a healthy diet can be applied to this population.

### 9.4. Omega-3 Polyunsaturated Fatty Acids (PUFAs)

Omega-3 polyunsaturated fatty acids (*n*-3 PUFAs) are known for their anti-inflammatory properties and are, therefore, of great interest in chronic inflammatory conditions such as COPD [167]. To date, there have been few studies examining the effects of *n*-3 PUFAs on health outcomes in COPD; however, the available studies have demonstrated positive effects on exercise tolerance [168,169,170,171], inflammation [170,171], and quality of life [170]. These findings were supported in a study finding similar benefits in patients with COPD (GOLD stage I–IV) who were already consuming *n*-3 PUFA supplementation [172]. Despite these improvements, the mechanisms linking *n*-3 PUFAs and the observed benefits in COPD are mostly unknown. It is known that *n*-3 PUFAs may increase the concentration of anti-inflammatory mediators and decrease the expression of adhesion molecules [173], which may be beneficial for patients with COPD. Additionally, *n*-3 PUFAs may exert multiple cardiovascular benefits, including reducing the risk of hypertension and coronary heart disease [174,175]. Indeed, current recommendations by the American Heart Association [175] and the American Dietetic Association along with the Dietitians of Canada [176] support a daily intake of *n*-3 PUFAs to observe health benefits, particularly due to the frequent low consumption of these products in many populations, including patients with COPD [172]. Of note, excessive consumption (>2 g per day from *n*-3 PUFAs supplementation) may be detrimental with potential toxic side effects [177]. In summary, despite the overall positive benefits associated with *n*-3 PUFAs, no recommendations can be made at this time for patients with COPD.

## 10. Practical Implications and Current Limitations

The 2024 GOLD report acknowledges the role that malnutrition and nutritional deficiencies have in COPD and associated comorbidities [38]. Notwithstanding, the report does not include dietary recommendations for the management of COPD, except for antioxidant supplementation [38], emphasizing the limited use of “food is medicine” in COPD. This is surprising, as there is ample literature to suggest the positive role of nutrition in the management of COPD [11], including information from the American Lung Association [178] and the European Respiratory Society [179]. This omission further highlights the lack of adequate nutritional research for COPD management, particularly emphasized by advancements achieved in other pathologies with clearer dietary recommendations [180,181,182] and even nutritional guidelines specific for the care of the patient after diagnosis [183]. Potential barriers to nutritional management may be related to the frequent lack of registered dieticians as part of the team caring for patients with COPD. Additionally, limited insurance coverage, challenges with transportation, or socioeconomic status will likely confound the difficulties in assessing and implementing adequate nutritional strategies in this population. It is also important to note that there is a significant lack of knowledge regarding how nutritional supplementation may interact with standard-of-care treatment for COPD. Considering that specific pharmacokinetics and pharmacodynamics differ between medications within a general subclass (i.e., different brands of short-acting beta agonists), this is an important consideration to evaluate when assessing the safety of nutritional supplements in this population. Finally, we must also consider that most of the current findings have been observed in demographically similar populations, and further studies are needed in diverse cohorts that are more representative samples of the global population affected by COPD.

## 11. Conclusions

COPD represents a highly prevalent condition, impacting more than 212 million people worldwide. Despite the prevalence and increased morbidity and mortality associated with this disease, there is a paucity of effective therapies that can treat, prevent, or even slow down the progression of this disease. Beyond pharmacological treatments, different preventative strategies have been explored, including the role of nutrition (Table 1). The increased consumption of fruits, fiber, vitamins, and/or iron, among other aspects, seems to exert beneficial effects in people with COPD, although further interventional studies are needed to establish clear guidelines in this population. Considering that many of these strategies can be applied in an affordable and accessible way, creating awareness about the connection between food and health is essential to truly maximize the therapeutic benefit for this population.

**Table 1:** The relationship between diet and nutritional supplements in the prevention and management of COPD.

Which Dietary and Nutritional Supplements May Be Beneficial for Patients with COPD?			
Diet and/or Nutritional Supplement	Risk of COPD Development?	Intake Has Potential Benefit Once COPD Diagnosis is Established?	Specific Dosage
Meat	↑		<75 g/week
Fruits and Vegetables	↓	✔	
Fiber	↓	✔	≥26.5 g/day
Vitamin D	↑ (deficiency)	✔	Serum 25(OH)D levels ≥ 55 nmol/L
Vitamin C	?	✔	400–1000 mg daily
Iron	↑ (deficiency)	✔	Intravenous ferric carboxymaltose
Nitrate	?	✔	BRJ with 12.9 mmol of nitrate twice weekly
Heavy Alcohol Consumption	↑		
Polyphenols	↓	✔	
Prudent Diet	↓		
Western Style Diet	↑		
DASH Diet	↓		
Mediterranean Diet	?		
*n*-3 PUFAs	?	✔	

↑ = increased risk. ↓ = decreased risk. ✔ = beneficial. ? = evidence inconclusive. nmol = nanomolar. mg = milligrams. BRJ = beetroot juice. mmol = millimoles. DASH = Dietary Approaches to Stop Hypertension. *n*-3 PUFAs = omega-3 polyunsaturated fatty acids.

## Data Availability

The original contributions presented in the study are included in the article, further inquiries can be directed to the corresponding authors.
